# Mutual Information and Information Gating in Synfire Chains

**DOI:** 10.3390/e20020102

**Published:** 2018-02-01

**Authors:** Zhuocheng Xiao, Binxu Wang, Andrew T. Sornborger, Louis Tao

**Affiliations:** 1 Department of Mathematics, University of Arizona, Tucson, AZ 85721, USA; 2 Center for Bioinformatics, National Laboratory of Protein Engineering and Plant Genetic Engineering, School of Life Sciences, Peking University, Beijing 100871, China; 3 Yuanpei School, Peking University, Beijing 100871, China; 4 Information Sciences, CCS-3, Los Alamos National Laboratory, Los Alamos, NM 87545, USA; 5 Department of Mathematics, University of California, Davis, CA 95616, USA; 6 Center for Quantitative Biology, Peking University, Beijing 100871, China

**Keywords:** pulse-gating, channel capacity, neural coding, feedforward networks, neural information propagation

## Abstract

Coherent neuronal activity is believed to underlie the transfer and processing of information in the brain. Coherent activity in the form of synchronous firing and oscillations has been measured in many brain regions and has been correlated with enhanced feature processing and other sensory and cognitive functions. In the theoretical context, synfire chains and the transfer of transient activity packets in feedforward networks have been appealed to in order to describe coherent spiking and information transfer. Recently, it has been demonstrated that the classical synfire chain architecture, with the addition of suitably timed gating currents, can support the graded transfer of mean firing rates in feedforward networks (called synfire-gated synfire chains—SGSCs). Here we study information propagation in SGSCs by examining mutual information as a function of layer number in a feedforward network. We explore the effects of gating and noise on information transfer in synfire chains and demonstrate that asymptotically, two main regions exist in parameter space where information may be propagated and its propagation is controlled by pulse-gating: a large region where binary codes may be propagated, and a smaller region near a cusp in parameter space that supports graded propagation across many layers.

## 1. Introduction

Faithful information transmission between neuronal populations is essential to computation in the brain. Correlated spiking activity has been measured experimentally between many brain areas [1,2,3,4,5,6,7,8]. Experimental and theoretical studies have shown that synchronized volleys of spikes can propagate within cortical networks and are thus capable of transmitting information between neuronal populations on millisecond timescales [9,10,11,12,13,14,15,16,17]. Many such mechanisms have been proposed for feedforward networks [14,15,17,18,19,20,21,22,23]. Commonly, mechanisms use transient synchronization to provide windows in time during which spikes may be transferred more easily from layer to layer [14,15,17,18,19,20,21,22].

For instance, the successful propagation of synchronous activity has been identified in “synfire chains” [18,19,21,24,25], wherein volleys of transiently synchronous spikes can be propagated through a predominantly excitatory feedforward architecture. Studies have shown that synchronous spike volleys can reliably drive responses through the visual cortex [13] with the temporal precision required for neural coding [26,27]. However, only sufficiently strong stimuli can elicit transient spike volleys that can successfully propagate through the network, and the waveform of spiking tends to an attractor with a single, fixed amplitude [21,28]. Thus, it is not possible to transfer graded information in the amplitudes of synchronously propagating spike volleys in this type of synfire chain.

Nonetheless, recent work has shown that synfire chains may be used as a pulse-gating mechanism coupled with a parallel “graded” chain (“synfire-gated synfire chain”—SGSC) to transfer arbitrary firing-rate amplitudes (graded information) through many layers in a feedforward neural circuit [14,15,16,17]. The addition of a companion gating circuit additionally provides a new mechanism for controlling information propagation in neural circuits [14,15,16,17]. Within a feedforward network, graded-rate transfer manifests as approximately time-translationally invariant spiking probabilities that propagate through many layers (guided by gating pulses) [17]. Using a Fokker–Planck (FP) approach, it has been demonstrated that this time-translational invariance arises near a cusp catastrophe in the parameter space of the gating current, synaptic strength and synaptic noise [17].

While many researchers have studied the dynamics of activity transmission in feedforward networks [21,23,25,29], few have examined these networks as information channels (however, see [30]). Shannon information [31,32] provides a natural framework with which to quantify the capacity of neural information transmission, by providing a measure of the correlation between input and output variables.

In particular, mutual information (MI), and measures based on MI, can be used to evaluate the expected reduction in entropy, for example, of the input, from the measurement of the output. Much work has focused on estimating probability distributions from spiking data (see, e.g., [33,34,35,36,37,38]). At the circuit level, studies have shown that MI is maximized in balanced networks (in cortical cultures, rats and macaques) that admit neuronal avalanches [39,40]. Furthermore, by examining the effects of gamma oscillations on MI in infralimbic and prelimbic cortex of mice, it has been shown that gamma rhythms enhance information transfer by reducing noise and signal amplification [7].

Theoretically, maximizing MI has been used to find nonlinear “infomax” networks [41] that can find statistically independent components capable of separating features in the visual scene [42]. MI has been used to assess the effectiveness and precision of population codes [43]. By combining decoding and MI, one can extract single-trial information from population activity and, at the same time, a quantitative estimate of how each neuron in the population contributes to the internal representation of the stimulus [44]. Furthermore, information-theoretic measures such as MI have been used on large-scale measurements of brain activity to estimate connectivity between different brain regions [45].

Here we consider information propagation properties in feedforward networks, and in particular, examine MI of mean firing-rate transfers across many layers of an SGSC neural circuit. We make use of an FP model to describe the dynamic evolution of membrane-potential probability densities in a pulse-gated, feedforward, integrate-and-fire (I&F) neuronal network [17]. We investigate the efficacy of information transfer in the parameter space near where graded mean firing-rate transfer is possible. We find that MI can be optimized by adjusting the strength of the gating (gating current), the feedforward synaptic strength, the level of synaptic noise, and the input distribution. Furthermore, our results reveal that via the coordination of pulse-gating and synaptic noise, a graded channel may be transformed into a binary channel. Our results demonstrate a wide range of possible information propagation choices in feedforward networks and the dynamic coding capacity of SGSCs.

## 2. Materials and Methods

We use a neuronal network model consisting of a set of j=1,⋯,M populations, each with i=1,⋯,N excitatory, current-based, I&F point neurons whose membrane potential, $V_{i,j}$, and (feedforward) synaptic current, $I_{i,j}^{\mathrm{ff}}$, are described by

(1a) $$\begin{array}{ccc} \frac{d}{dt}V_{i,j}^{} & = & -g_{L}(V_{i,j}^{}-V_{R})+I_{g,j}+I_{i,j}^{\mathrm{ff}} \end{array}$$

(1b) $$\begin{array}{ccc} \tau \frac{d}{dt}I_{i,j}^{\mathrm{ff}} & = & -I_{i,j}^{\mathrm{ff}}+\left\{\begin{array}{cc} \frac{S}{pN}\sum_{k}p_{jk}\sum_{l}\delta \left(t-t_{j-1,k}^{l}\right), & j>1\\ A\delta (t), & j=1 \end{array}\right. \end{array}$$

where $V_{R}$ is the reset voltage, τ is the synaptic timescale, *S* is the synaptic coupling strength, $p_{jk}$ is a Bernoulli distributed random variable and $p=\langle p_{jk}\rangle$ is the mean synaptic coupling probability. The *l*th spike time of the *k*th neuron in layer j−1 is determined by $V\left(t_{j-1,k}^{l}\right)=V_{Th}$, that is, when the neuron reaches the threshold. The gating current, $I_{g,j}$, is a white-noise process with a square pulse envelope, θ(t−(j−1)T)−θ(t−jT), where θ is a Heaviside theta function and *T* is the pulse length [14] of pulse height $I_{g}$ and variance $\sigma_{0}^{2}$. We note that with the j=1 equation, an exponentially decaying current is injected into population 1, providing graded synchronized activity that subsequently propagates downstream through populations j=2,⋯,M.

Assuming the spike trains in Equation (1b) to be Poisson-distributed, the collective behavior of this feedforward network may be described by the FP equations:

(2a) $$\begin{array}{ccc} \frac{\partial }{\partial t}\rho_{j}\left(V,t\right) & = & -\frac{\partial }{\partial V}J_{j}\left(V,t\right) \end{array}$$

(2b) $$\begin{array}{ccc} \tau \frac{d}{dt}I_{j}^{\mathrm{ff}} & = & -I_{j}^{\mathrm{ff}}+\left\{\begin{array}{cc} Sm_{j-1}, & j>1\\ A\delta (t), & j=1 \end{array}\right. \end{array}$$

(While the output spike-train of a single neuron in general does not obey Poisson statistics, the spike train obtained from the summed output of all neurons in a single population does obey these statistics asymptotically for a large network size *N*. In the case of pulse-gating, the summed output spikes of a single population tend to a time-dependent Poisson process.) These equations describe the evolution of the probability density function (PDF), $\rho_{j}\left(V,t\right)$, in terms of the probability density flux, $J_{j}\left(V,t\right)$, the mean feedforward synaptic current, $I_{j}^{\mathrm{ff}}\equiv \langle I_{i,j}^{\mathrm{ff}}\rangle$, and the population firing rate, $m_{j}$. For each layer *j*, the probability density function gives the probability of finding a neuron with membrane potential $V\in (-\infty ,V_{Th}]$ at time *t*.

The probability density flux is given by

$$J_{j}\left(V,t\right)=\left(\left[-g_{L}\left(V-V_{R}\right)+I_{g}+I_{j}^{\mathrm{ff}}\right]-\sigma_{j}^{2}\frac{\partial }{\partial V}\right)\rho_{j}\left(V,t\right)\;$$

where $I_{g}$ indicates the mean gating current. The effective diffusivity is

(3) $$\sigma_{j}^{2}=\sigma_{0}^{2}+\frac{1}{2}\frac{S^{2}}{pN}m_{j-1}\left(t\right)\;$$

In the simulations reported below, we have taken N→∞, thus ignoring the second term in Equation (3) (i.e., ignoring diffusion due to finite size effects). The population firing rate is the flux of the PDF at threshold:

(4) $$m_{j}\left(t\right)=J_{j}\left(V_{Th},t\right)\;$$

The boundary conditions for the FP equations are

(5) $$\begin{array}{ccc} J_{j}\left(V_{R}^{+},t\right) & = & J_{j}\left(V_{Th},t\right)+J_{j}\left(V_{R}^{-},t\right)\; \end{array}$$

(6) $$\begin{array}{ccc} \rho_{j}\left(V_{R}^{+},t\right) & = & \rho_{j}\left(V_{Th},t\right)+\rho_{j}\left(V_{R}^{-},t\right)\; \end{array}$$

and

(7) $$\rho_{j}\left(V=-\infty ,t\right)=0\;$$

To efficiently obtain solutions to the FP equations [17], we have used an approximate Gaussian initial distribution, $\rho_{j}\left(V,t+jT\right)=\left(1/P\right)\exp\left(-{\left(V-\mu \left(t\right)\right)}^{2}/2\sigma^{2}\right)$, with width σ and mean μ(t), where $P=\int_{-\infty }^{V_{\mathrm{Th}}}\rho_{0}\left(V,0\right)$ is a normalization factor that accounts for the truncation of the Gaussian at threshold, $V_{\mathrm{Th}}$. At the onset of gating, the distribution is advected toward the voltage threshold, $V_{\mathrm{Th}}$, and the population starts to fire. The advection neglects a small amount of firing due to a diffusive flux across the firing threshold; thus the fold bifurcation occurs at a slightly larger value of synaptic coupling, *S*, for this approximation, relative to numerical simulations [17]. Because the pulse is fast, neurons only have time to fire once (approximately). Thus, we neglect the re-emergent population at $V_{R}$, which does not have enough time to advect to $V_{\mathrm{Th}}$ and therefore does not contribute to firing during the transient pulse.

Using this approximation, Equation (2a) gives rise to $\dot{\mu }=-g_{L}\left(\mu -V_{R}\right)+I^{g}+I_{u}^{\mathrm{ff}}$, where $\sigma^{2}=\;\sigma_{0}^{2}/g_{L}$, with upstream current $I_{u}^{\mathrm{ff}}=Ae^{-t/\tau }$. Setting $V_{\mathrm{Th}}=1$, this integrates to

(8) $$\mu \left(t\right)=\mu_{0}e^{-g_{L}t}+\frac{I^{g}}{g_{L}}\left(1-e^{-g_{L}t}\right)+\frac{A}{\frac{1}{\tau }-g_{L}}\left(e^{-g_{L}t}-e^{-t/\tau }\right)$$

and from Equation (4), we have

(9) $$m\left(t\right)={\left[\left(-g_{L}\mu \left(t\right)+I^{g}+Ae^{-t/\tau }\right)\frac{1}{P}e^{-{\left(1-\mu \left(t\right)\right)}^{2}/2\sigma^{2}}\right]}^{+}\;$$

which, from Equation (2b), results in a downstream synaptic current at t=T:

(10) $$I_{d}^{\mathrm{ff}}=Se^{-T/\tau }\int_{0}^{T}e^{t/\tau }m\left(t\right)\frac{dt}{\tau }\;$$

After the gating pulse terminates, the current decays exponentially. This decaying current feeds forward and is integrated by the next layer. For an exact transfer, $I_{d}^{\mathrm{ff}}\left(S,I^{g},A,T\right)=A$.

To compute MI, we generated a distribution of upstream current amplitudes $\left\{I_{u}\right\}$. These were typically within or near the range of fixed points of the map $I_{d}^{\mathrm{ff}}\left(S,I^{g},A,T\right)=A$. Using Equations (8)–(10), we generated a distribution of downstream current amplitudes $\left\{I_{d}\right\}$ with $\left\{I_{u}\right\}$ as initial values. The joint probability distribution $p(I_{u},I_{d})$ was estimated by forming a two-dimensional histogram with ΔI=0.3/s. MI, $I\left(I_{u};I_{d}\right)=H\left(I_{u}\right)+H\left(I_{d}\right)-H\left(I_{u},I_{d}\right)$, was computed (in bits) with $H\left(x\right)=-\sum_{x}p\left(x\right)\log_{2}\left(p\left(x\right)\right)$ and $H\left(x,y\right)=-\sum_{x,y}p\left(x,y\right)\log_{2}\left(p\left(x,y\right)\right)$, where the marginal probabilities $p(I_{u})$ and $p(I_{d})$ were computed from the histogram.

## 3. Results

We consider the transfer of spiking activity between two successive layers of a pulse-gated feedforward network. The spiking activity of feedforward propagation quickly converges to a stereotypical firing-rate waveform with arbitrary amplitudes (within a certain range) and with associated dynamics of the membrane potential PDF, ρ(V,t), for each layer. This waveform essentially represents a volley of spikes that propagate downstream within the network. Furthermore, the temporal evolution of the first two moments (i.e., mean and variance) of ρ(V,t) suffice to capture the dynamics during pulse-gating [17]. Therefore, as [17] showed, the population dynamics across a large region of parameter space can be mapped by using a Gaussian approximation to the membrane potential PDF (see Materials and Methods), revealing the bifurcation structure underlying a cusp catastrophe.

**Figure 1:**
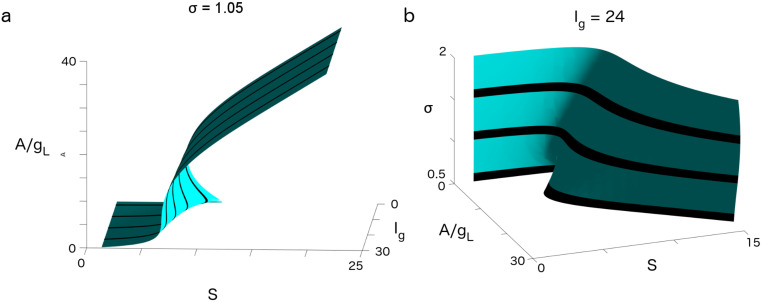
Cusp enabling graded and binary propagation in a synfire-gated synfire chain (SGSC). (**a**) View for fixed noise, σ=1.05, as a function of firing-rate amplitude, $A/g_{L}$; synaptic strength, *S*; and gating current, $I_{g}$. (**b**) View for fixed gating current, $I_{g}=24$, as a function of $A/g_{L}$, *S*, and σ. Plotted in both panels are zeros of the function $I(A,I_{g},\sigma )-A$ (the difference between input and output firing-rate amplitudes in a given layer). This function becomes zero at a fixed point. At a fixed point, a firing-rate amplitude propagates exactly from one layer to the next. When this function is small but non-zero, the firing-rate amplitude only changes slowly as it propagates. We note that the cusp is high-dimensional and can be viewed in various projections. Graded propagation (approximately exact amplitude propagation) is enabled as a result of ghost (slow) dynamics near the cusp, such that the firing-rate amplitude *A* varies only slowly from layer to layer. At the cusp, an approximate line attractor exists where an input firing rate in a given layer changes only slowly as it propagates downstream. Away from the cusp, firing rates rapidly approach attractors. For many parameter regions, there are two attractors giving rise to the propagation of a binary code.

We first examined the cusp catastrophe in SGSC systems and its role in shaping the transfer of mean firing rates. Because of the existence of stable, attracting, translationally invariant firing-rate waveforms, we could capture and understand the dynamics in the SGSC system as an iterated map describing the firing-rate amplitude as it changed between successive network layers. Figure 1 shows two cases of the fixed points of this iterated map. Figure 1a plots the firing-rate amplitude, *A*, at the fixed points at the end of the pulse-gating period as a function of the strength of the gating current, $I_{g}$, and synaptic coupling strength, *S*, for fixed σ=1.05. For small $I_{g}$, there existed a range in *S* where the system was bistable; however, as we increased the strength of the gating, the region of bistability (two stable attracting solutions with an unstable solution in between) disappeared at a cusp where the manifold of the fixed point was nearly vertical. It was shown in [17] that near this cusp, the slow dynamics along the unstable manifold of the unstable fixed point allow for the nearly graded transfer of firing rates between successive layers, giving rise to an approximate line attractor in the amplitude of the output firing rates. Figure 1b shows that this cusp catastrophe also existed at fixed gating current $I_{g}=24$ as we varied both the feedforward synaptic coupling strength *S* and the variance of the initial PDF, ρ(V,t=0), just before pulse-gating. As the variance, $\sigma^{2}$, was increased, the bistable region in *S* also disappeared.

**Figure 2:**
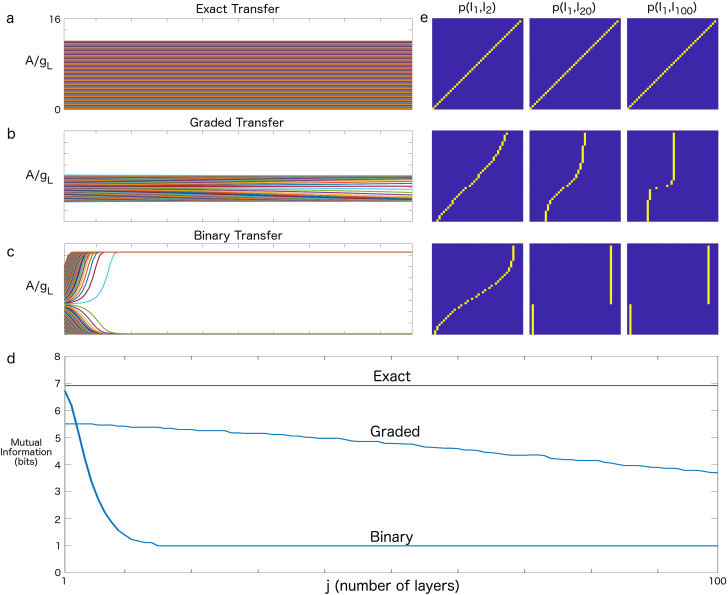
Mutual information (MI) for transfers across a 100 layer synfire-gated synfire chain (SGSC). (**a**) Exact transfer: Firing-rate amplitudes for theoretically perfect transfer. The range of $A/g_{L}$ is the same for (**a**–**c**). Amplitudes transfer exactly from one layer to the next (j=1,⋯,100). This practically unattainable communication mode is shown for comparison and attains the maximum possible information transfer (MI; see (**d**)) as a function of the layer. (**b**) Graded transfer: Firing-rate amplitudes from approximate Fokker–Planck (FP) solutions in a multi-layer SGSC. Parameters: $I_{g}=24.03$; S=7.001; σ=1.0496. We note that as the number of layers through which the initial amplitudes propagate increases, the amplitudes drift slowly away from an unstable attractor in the center of the amplitude distribution towards stable attractors at the sides of the distribution. (**c**) Binary transfer: Firing-rate amplitudes from approximate FP solutions in a multi-layer SGSC. Parameters: $I_{g}=5.0$; S=7; σ=0.6. For these parameters, the unstable attractor rapidly repels the amplitudes toward stable attractors on either side, resulting in binary transfer. Binary transfer is extremely stable in the SGSC. (**d**) MI as a function of layer, *j*, for each of the above types of channel (**a**–**c**). (**e**) The joint probabilities, $p(I_{1},I_{2})$, $p(I_{1},I_{20})$, and $p(I_{1},I_{100})$, from which the MI was computed for exact, graded and binary transfer. Here, the exact case is as would be expected. The graded case shows a gradual deformation away from the diagonal that approaches binary transfer asymptotically. The binary case rapidly approaches the propagation of only two states. We note that in much of the parameter space, the approach to binary is much faster than that shown here. We used these parameters so that the transition was slower and therefore more evident.

Next, we examine the evolution of MI through the network. Figure 2 shows the transmission of the mean firing rate, $A/g_{L}$, in a pulse-gated feedforward network, as well as the associated MI for three types of channels. Figure 2a plots the firing rate for an idealized exact transfer across many layers (j=1,⋯,100), where we utilized a thresholded linear f–I curve to model each layer [14]. In this toy model, the transfer was exact and the mean firing rate propagated indefinitely without change. Figure 2b shows the propagation of firing rates for an SGSC situated near the cusp of Figure 1a. The long-term propagation of the initially uniform input firing rates revealed the dynamical structure near the cusp, namely, two stable, attracting firing rates (top and bottom) with an unstable saddle in between. Because of the slowness of the dynamics along the unstable manifold of the saddle, the transfer of mean firing rates through the network was approximately graded for many layers (j≈ 1–30). Furthermore, this transfer of the mean firing rates was order preserving (i.e., the relative ordering of amplitudes was maintained) across many more layers (*j* up to 100). Figure 2c demonstrates the propagation of firing rates for a binary transfer, where the unstable saddle was strongly repelling; for most initial conditions, the rates converged to one of two rates within 10–15 layers. To investigate the effects of the SGSC dynamics on information propagation, we computed the MI for each of the three cases. Figure 2d demonstrates the effect of the SGSC dynamics on information propagation. In the exact transfer case, the MI between the input and each layer remained constant and represented an upper bound on the information transfer. In the binary transfer case, the MI quickly decayed to 1 bit, but, as for the exact case, was stable over long timescales. Near the cusp, where the firing-rate transfer was approximately graded, the MI decayed slowly, so that even after j=100 transfers, the channel retained almost 4 bits of information. In Figure 2e, the joint probabilities from which the MI was computed for representative layers are plotted. For the exact case, the distribution is always along the diagonal. The fast transition from diagonal to binary is evident for binary transfers, and a slow transition from diagonal to binary is seen for the graded case.

**Figure 3:**
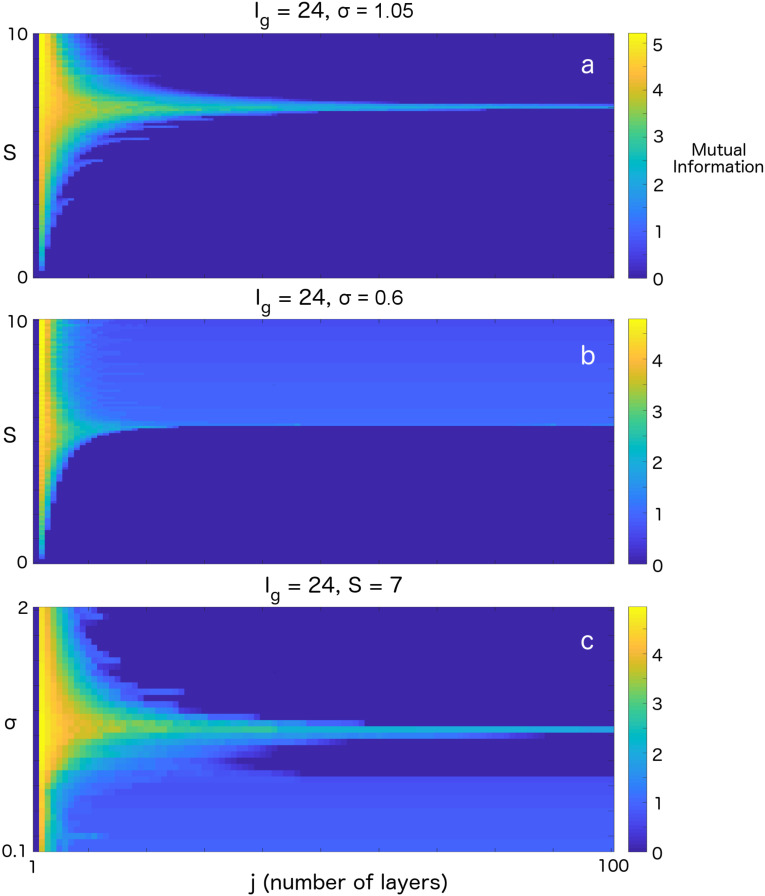
Mutual information (MI) for parameters supporting graded and binary codes. (**a**) MI propagation across 100 layers for $I_{g}=24$, σ=1.05 and *S* ranging from 0 to 10. We note that graded transfer allows high values of MI to propagate across 100 layers at S≈7. (**b**) MI propagation across 100 layers for $I_{g}=24$, σ=0.6 and *S* from 0 to 10. We note that above S≈5, there is a large range of *S* for which binary propagation (MI of 1 bit; see colorbar) is supported. (**c**) MI propagation for $I_{g}=24$, S=7 and σ from 0.1 to 2. Here, both graded and binary information propagate depending on the value of σ.

An FP analysis of the SGSC system reveals four important system parameters: strength of the gating current, $I_{g}$; strength of the feedforward synaptic coupling, *S*; mean membrane potential distribution at the beginning of pulse-gating, $\rho_{j}\left(V,t=jT\right)$; and variance of the synaptic current, $\sigma^{2}$ (see Materials and Methods). Figure 3 shows where graded and binary codes are supported. Figure 3a,b plots the MI as a function of *S* and the network layer (which is equivalent to propagation time) for fixed $I_{g}$, σ and $\rho_{j}\left(V,t=jT\right)$ (which equals 0 for all panels). As we can see, for both cases, there exists an optimal *S* for which MI (and approximate graded activity) can be maintained for many layers. As we move away from this optimal *S*, we quickly go into a binary coding regime (MI=1). Figure 3c demonstrates that similar qualitative behavior can be obtained by varying σ.

**Figure 4:**
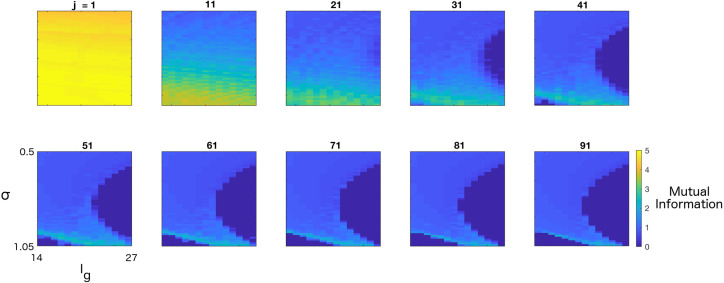
Regions of parameter space supporting graded and binary information propagation. Visualization of mutual information (MI) for σ between 0.5 and 1.05 and $I_{g}$ between 14 and 27 at successive layers *j*; σ and $I_{g}$ axes as denoted in the lower left panel are the same for all panels. Colorbar for lower right panel is the same for all panels. We note that across a few layers, MI remains high for a large region of parameter space, but by j=21, only binary and graded codes persist. By j=91, it is seen that a large region of parameter space supports binary propagation. Along a thin diagonal line at the bottom of the binary region, corresponding to the location of the cusp, graded information may be transferred.

Out of the four system parameters, it is easiest to manipulate either the gating current or the variance of the synaptic current. Both can be viewed as controls independent of the SGSC system. Therefore, in Figure 4, we examine the evolution of MI as a function of $I_{g}$ and σ through the network. As we expect from our results thus far, large regions of parameter space support binary coding; however, there is a thin line that materializes towards the bottom of the binary region that corresponds to the location of the cusp, where MI can be maintained at high levels (MI>3) through many layers. We note that asymptotically, as j→∞, MI approaches 1 (a binary channel) because of the existence of two attractors, even near the cusp.

## 4. Discussion

Although coherent activity has now been measured in many regions of the mammalian brain, the precise mechanism and the extent to which the brain can make use of synchronous spiking activity to transfer information have remained unclear. Many mechanisms have been proposed for information transfer via transiently synchronous spiking that relies on oscillations and gating [18,20,22,46,47,48,49,50,51,52]. These mechanisms make use of the fact that coherent input can provide temporal windows during which spiking activity may be more easily transferred between sending and receiving populations. However, from the theoretical perspective, how MI and other measures of communication capacity can be related to the underlying neuronal network architecture and the emergent network dynamics have remained unexplored.

Here we study the capacity for information transfer of feedforward networks by examining the evolution of mutual information through many layers of an SGSC system. Previous work has showed that by introducing suitably timed pulses, graded information could be transferred and controlled in a feedforward excitatory neuronal network [14,15,16,17]. In this context, pulse-gating has allowed us to understand information propagation as an iterated map. FP analysis of this map has enabled the identification of a cusp catastrophe in the relevant parameter space. Our results here demonstrate that the dynamics of the SGSC system naturally give rise to two different types of channel (as measured by MI). Large regions of parameter space support binary coding by using transiently synchronous propagation of high and low firing rates. (In the classical synfire chain case, the low firing-rate state is a silent state. In the SGSC case, as a result of the gating current, it is small, but non-zero.) In the binary regime, the distance between low and high firing rates is much larger than the variance of the distributions; therefore propagation is stable. Furthermore, by systematically varying the relevant SGSC system parameters, we were able to optimize graded-rate transfer near the fold of a cusp catastrophe, which enabled us to maintain a relatively high MI through many layers.

Evidence exists for graded information coding in visual and other cortices [53], and there is some evidence for binary coding in the auditory cortex [54]; sparse coding mechanisms for its use have been put forward [55,56]. Luczak et al. [51] have argued that spike packets and stereotypical and repeating sequences (similar to sequences observed in implementations of information processing algorithms in graded-transfer SGSCs [15]) underlie neural coding. More recently, Piet et al. [57] have used attractor networks to model frontal orienting fields in rat cortices to argue that bistable attractor dynamics can account for the memory observed in a perceptual decision-making task. Indeed, in many decision-making tasks, cortical activity appears to be holding graded information in *working memory*, before a *decision* forces the activity into a binary code [58]. Therefore, it is of interest that both graded and binary pulse-gated channels are supported by the SGSC mechanism and that it is fairly simple and rapid to convert a graded code to a binary code.

Examining the structure of the cusp catastrophe in the parameter space, it appears that in general, weaker $I_{g}$ and higher *S* are correlated with bistability, while stronger $I_{g}$ can support graded information transfer at lower *S* (see Figure 1a); at the same time, for fixed $I_{g}$, a lower σ tends to bistable states (see Figure 1b).

In previous theoretical studies of graded propagation and line attractors, it has been shown that some fine tuning of system parameters is required [59,60,61]. However, graded propagation and line attractors have been observed in many areas of the brain (see, e.g., [62,63,64,65]). Our results here demonstrate that an important consideration for understanding graded propagation is the depth of the circuit being used to propagate the information. That is, graded information propagation circuits with a depth of 20 to 30 layers are not particularly fine-tuned. In this depth range, about 1/10th of the parameter space (e.g., *S* or σ; see Figure 3, top and bottom plots) is capable of propagating graded information (with relatively high MI). As the depth of the circuit grows, j→∞, MI approaches 1 bit, and hence graded propagation in deep circuits is not possible (with an SGSC with parameters near the cusp).

A clear advantage of a graded information channel is that a vector of high-resolution graded information (resolution $2^{n}$, where *n* is the number of bits of MI—up to 32 levels of resolution in the graded channel shown in Figure 2) could be rapidly processed in a network with linear synaptic connectivity. Thus, synaptic processing such as Gabor transforms, seen in the visual cortex, would most naturally operate on graded information, rapidly reducing the dimension of and orthogonalizing input data. However, the stable processing of information through deep and complicated neural logic circuits would take better advantage of binary channels. Here, essentially exact pulse-gated binary transformations and decisions [15] can make use of the attractor structure of the channel to reduce noise and maintain discrete states. In order to make use of high-bandwidth graded processing, at some point in a neural circuit, graded information would need to be transformed to binary information. Mechanisms to do this are beyond the scope of this paper, but they could make use of dimension-reducing transforms on the input (e.g., Gabor transform) and subsequent digitization of the data subspace.

Finally, we note that the symmetric nature of the binary SGSC channel may be an advantage for logic circuit gating, as either of the parallel synfire chains can operate as information or the gate [15]. Finally, it should be remembered that with a stable binary code, binary digit coding can be constructed, effectively increasing the information resolution propagating in a binary circuit.

## 5. Conclusions

We have performed an investigation of the communication capabilities of SGSCs using the metric of MI. The main conclusion of this investigation is that SGSCs sustain two types of channel: the first is binary transfer (MI = 1, in bits), which is supported for a wide range of parameters. For circuit depths of up to 30–40 layers, in a narrower range of parameters, graded transfer is also supported. A secondary conclusion is that, because of the dependence of MI on the depth of a circuit in some parameter regimes, circuit depth should be taken into account when considering the communication capacity of a neural circuit.
